# Interfacial Tuning of Sulfohalide Electrolytes by LiBF_4_ for Stable Lithium Metal Batteries

**DOI:** 10.3390/molecules31132313

**Published:** 2026-07-01

**Authors:** Peng Tang, John Prochest Kachenje, Zhengle Xiang, Dachun Wang, Yanyi Tao, Peng Yang, Huihui Li, Xiaoping Qin, Song Qing, Wei Cao, Qinyu Chen, Yongmin Wu, Haiyang Tian

**Affiliations:** 1 School of Chemical Engineering, Sichuan University of Science and Engineering, Zigong 643000, China; johnkachenje97@gmail.com (J.P.K.); 18081294612@163.com (Y.T.); 15228402327@163.com (P.Y.); janet.lihuihui@gmail.com (H.L.); 13568322767@163.com (X.Q.); 18200331076@163.com (S.Q.); 13198440339@163.com (W.C.); chenailu215617@outlook.com (Q.C.); haiyangtian@suse.edu.cn (H.T.); 2 Clean Energy Branch of China National Offshore Oil Corporation Energy Technology & Services Co., Ltd., 293 Tangubohaishiyou Road, Tianjin 300450, China; 13920080354@126.com (Z.X.); wangdch2@cnooc.com.cn (D.W.); 3 State Key Laboratory of Space Power-Sources Technology, Shanghai Institute of Space Power Sources, 2965 Dongchuan Road, Shanghai 200245, China

**Keywords:** lithium metal batteries, solid-state electrolytes, LiBF_4_, sulfohalide, ionic conductivity, interfacial stability

## Abstract

Lithium metal batteries (LMBs) incorporating solid-state electrolytes (SSEs) promise high energy density and safety, yet their practical deployment is hindered by poor interfacial stability between SSEs and lithium metal anodes. Here we show that a simple incorporation of LiBF_4_ into the sulfohalide (Li_3_SCl) framework forms a mixture Li_3_SCl@LiBF_4_ (LSC@BF) SSE via a two-step solid-state synthesis, preserving a high room-temperature ionic conductivity of 4.32 × 10^−4^ S cm^−1^ with a low activation energy of 0.22 eV while fundamentally altering the interface. X-ray photoelectron spectroscopy and electron microscopy reveal that LiBF_4_ promotes the in situ formation of a mechanically robust, LiF-rich solid-electrolyte interphase at the SSE|Li interface. This LiF-rich layer effectively suppresses lithium dendrite growth and stabilizes the interface, enabling symmetric Li|LSC@BF|Li cells to achieve stable lithium plating/stripping for over 800 h at 0.2 mA cm^−2^. Cross-sectional post-mortem imaging confirms a dense, void-free interface without dendrite penetration. Our work demonstrates that LiBF_4_ incorporation offers a simple, scalable strategy to simultaneously maintain high ionic conductivity and resolve interfacial instability in sulfohalide SSEs for high-performance LMBs.

## 1. Introduction

The development of solid-state electrolytes (SSEs) with desirable properties for lithium metal batteries (LMBs) offers a realistic pathway beyond conventional liquid electrolyte, promising higher energy density and intrinsic safety [1,2]. While significant research has been devoted to oxide, halide, LISICON, sulfide, and polymer-based SSEs, SSEs still face challenges in balancing crucial properties, particularly in achieving high conductivity while maintaining stability with a lithium metal anode [3,4,5]. Among inorganic SSEs, sulfide-based SSEs stand out for their exceptional room-temperature ionic conductivity (often >10^−3^ S cm^−1^), favorable mechanical compliance, and low grain-boundary resistance [5,6,7]. Yet, their practical application is crippled by a persistent Achilles’ heel: poor interfacial compatibility with bare lithium metal anodes. Sulfide SSEs are electrochemically reduced upon contact with Li, leading to an uncontrollably growing passivation layer, rising interfacial impedance [8,9], and ultimately resulting in the short-circuit of the cell [10], owing to their non-negligible electronic conductivity to facilitate the non-uniform Li deposition and propagation of lithium dendrites [11]. Numerous strategies have been pursued to tame this interface. Artificial protective coatings (oxides, LiPON, polymers) and Li-alloying with metals such as In, Au, Ag or Mg [12,13,14,15,16] can physically separate the SSE from Li. However, they add manufacturing complexity and cost, and many coating materials suffer from low ionic conductivity, constraining overall cell performance [17]. More recently, fluorination has emerged as a more chemically targeted approach: fluorine promotes the in situ formation of a LiF-rich solid-electrolyte interphase (SEI), which is both electronically insulating and mechanically robust, thereby suppressing further decomposition and dendrite growth [18,19,20]. Unfortunately, the strong electronegativity of fluorine typically reduces bulk ionic conductivity to prohibitive ≤10^−5^ S cm^−1^ at room temperature [21,22].

We recently showed that controlled fluorination of the sulfohalide Li_3_SCl (LSC) SSE can preserve high conductivity while improving interfacial stability, enabling stable cycling of symmetric Li cells for 580 h at 0.1 mA cm^−2^ [23]. Although promising, this lifetime remains insufficient for real-world applications. Here we demonstrate a markedly more effective route: the simple incorporation of LiBF_4_ into the LSC framework. LiBF_4_ is well known in liquid electrolytes for its oxidation stability and low interfacial charge-transfer resistance. In addition, LiBF_4_ is known to serve as an effective fluorine source, facilitating the sacrificial decomposition-induced formation of a LiF-rich SEI, which is critical for stabilizing the lithium metal interface [24,25,26]. However, its role in sulfide-based solid systems has been unexplored. We find that LiBF_4_ not only retains the high ionic conductivity of LSC (4.32 × 10^−4^ S cm^−1^) but also facilitates the in situ formation of a dense, LiF-dominated SEI that is far more stable than that obtained by direct fluorination. This synergy allows symmetric Li cells to operate for >800 h at 0.2 mA cm^−2^, which is a doubling of lifetime at double the current density, without dendrite penetration. By combining structural, transport and interfacial characterization, we unravel how BF_4_^−^ modulate the interface chemistry while leaving the bulk conduction pathway intact. Our work establishes LiBF_4_ incorporation as a scalable, one-step strategy to resolve the long-standing conductivity–stability trade-off in sulfohalide SSEs for high-performance LMBs.

## 2. Experimental Methods

### 2.1. Preparation of LSC and LSC@BF

**Figure 1:**
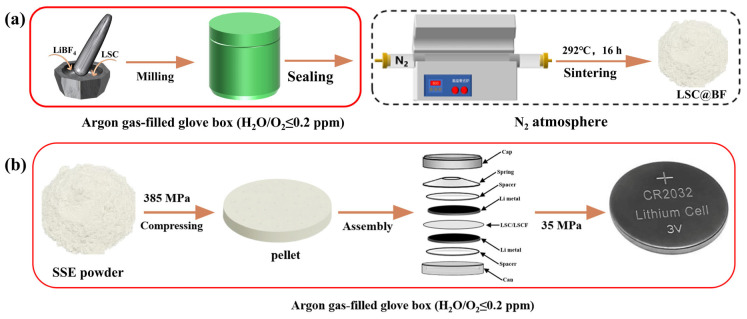
Schemes for (**a**) preparation of LSC and LSC@BF mixture SSEs, (**b**) symmetric cell preparation.

The Li_3_SCl@LiBF_4_ (LSC@BF) mixture was prepared via a two-step solid-state procedure. First, stoichiometric amounts of Li_2_S and LiCl (both 99.9% pure, bought from Shanghai Jiecai Biotechnology Co., Ltd. Shanghai, China) were mixed. The intermediate Li_3_SCl was prepared by firstly manual grinding in an agate mortar inside an argon-filled glove box (O_2_/H_2_O ≤ 0.2). This mixture was sealed in an aluminum oxide ampule, wrapped with a layer of thick, soft aluminum foil. Subsequently, it was sintered at 550 °C for 4 h under a nitrogen atmosphere, followed by slow quenching, in which the sample was left to cool naturally to room temperature inside the tube furnace before being transferred back into the glove box and ground into a fine crystalline powder (Figure 1a). In the second step, the LSC powder intermediate was stoichiometrically mixed with LiBF_4_, thoroughly ground and mixed in an agate mortar for 20 min. The new mixture was again packed into an aluminum oxide ampule and sealed with thick aluminum foil. A second annealing step was performed in the tube furnace at 292 °C for 16 h under nitrogen atmosphere to form the target product of LSC@BF. The same procedure was repeated to prepare LSC@BF mixture with different amounts of LiBF_4_ salt. Then, the corresponding powders were pressed into pellets (with a thickness of 1~1.2 mm and a diameter of 15 mm) and assembled into LMBs as shown in Figure 1b.

### 2.2. Material Characterizations

The phase composition and crystal structure of LSC and LSC@BF were analyzed using X-ray diffractometer (XRD, D2 phaser, Bruker, Karlsruhe, Germany) with Cu Kα radiation in a 2θ range from 10° to 90° at a scan rate 2°/min at room temperature. Field emission scanning electron microscope (FESEM, SEM5000, Oxford instruments, Oxford, UK) coupled with energy dispersive spectrometer (EDS, XFlash Detector 410-M, National Instrument Co., Ltd., Billerica, MA, USA) was used to observe the morphology and elemental distribution of as-prepared SSEs and post-cycling Li metal surface as well as the cross-section of the SSEs|Li interface. Furthermore, SEI chemical composition on the post-cycling Li metal was studied by X-ray photoelectron spectroscopy (XPS, K-Alpha, Thermo Fisher Scientific, Waltham, MA, USA).

### 2.3. Electrochemical Measurements

LSC and LSC@BF powders (200–300 mg) were pressed into pellets (15 mm diameter, 1–1.2 mm thickness) under 380 MPa at room temperature as shown in Figure S1. Each pellet was sandwiched between two stainless steel electrodes (15 mm diameter, 0.3 mm thickness) under 35 MPa for electrochemical impedance spectroscopy (EIS) measurements using a CHI760E potentiostat. The temperature was increased stepwise from room temperature to 80 °C in 10 °C increments. EIS spectra were recorded over a frequency range of 1 Hz to 10^6^ Hz with an amplitude of 5 mV.

The ionic conductivity (σ) of LSC and LSC@BF SSEs were calculated using $\sigma =L/R_{b}S$, where *R*_b_ is the bulk resistance (Ω), *L* (cm) and *S* (cm^2^) are the thickness and area of the pellets. The activation energy (*E*_a_) for the temperature-dependent ionic conductivity were derived from the Arrhenius equation, $\sigma_{\mathrm{ion}}\left(T\right)\;=\;A(T)\exp(-E_{a}/\mathrm{RT})$, where A(T) is the pre-exponential factor, *R* is the gas constant (*R* = 8.314 J mol^−1^ K^−1^), and *T* is the absolute temperature (K).

Furthermore, the electronic conductivity ($\sigma_{e}$) was measured using stainless steel symmetric cells with LSC or LSC@BF SSEs pellets. A direct current (DC) polarization of 0.1 V was applied, and the steady-state current was recorded. $\sigma_{e}$ was calculated from Ohm’s law, $\sigma_{e}=\;L\;/\;S\;\times \;I\;/\;V$, where *L* and S are the thickness (cm) and area (cm^2^) of the SSE pellet, and *I* (A) and *V* (V) are the steady-state current and voltage, respectively.

Lithium symmetric cells (CR203) were assembled to evaluate the cycling stability of LSC@BF when compared with its pristine LSC. Prior to assembly, both sides of the SSE pellet were wetted with a trace of 1 M LiTFSI (EC/DMC), then sandwiched between two pieces of lithium metal foils (with 14 mm diameter and 0.3 mm thickness). The plating/stripping performance of the symmetric cells were tested using Neware BTS (BTS-4000-5V10mA, Shenzhen PRC, Shenzhen, China) at room temperature.

For post-cycling electrode characterization, the Li|LSC|Li and Li|LSC@BF|Li symmetric cell was disassembled inside an argon-filled glove box (H_2_O < 0.1 ppm, O_2_ < 0.1 ppm) after 100 h of cycling at 0.1 mA cm^−2^. The lithium metal electrodes were carefully extracted and left to dry overnight in argon gas-filled glove box at room temperature. The dried electrodes were transferred to the XPS and FESEM instruments using a vacuum transfer vessel to avoid air exposure.

The Li|LSC|Li and Li|LSC@BF|Li symmetric cells EIS spectra used in analyzing the interfacial impedance were fitted using an equivalent circuit consisting of a resistor (R_1_) for bulk resistance, a resistor (R_2_) and a constant phase element (CPE_1_) in parallel for grain boundary and interfacial contributions, and another resistor (R_3_) with the second constant phase element (CPE_2_) for electrode polarization.

Cross-sectional imaging of the SSE|Li interface was performed on intact bilayer sample (SSE adherent to Li metal). After disassembly, the stack was cut with a sharp razor blade, and the freshly exposed cross-section was examined by FESEM without separating the two layers.

## 3. Results and Discussion

### 3.1. Phase and Structural Analysis

**Figure 2:**
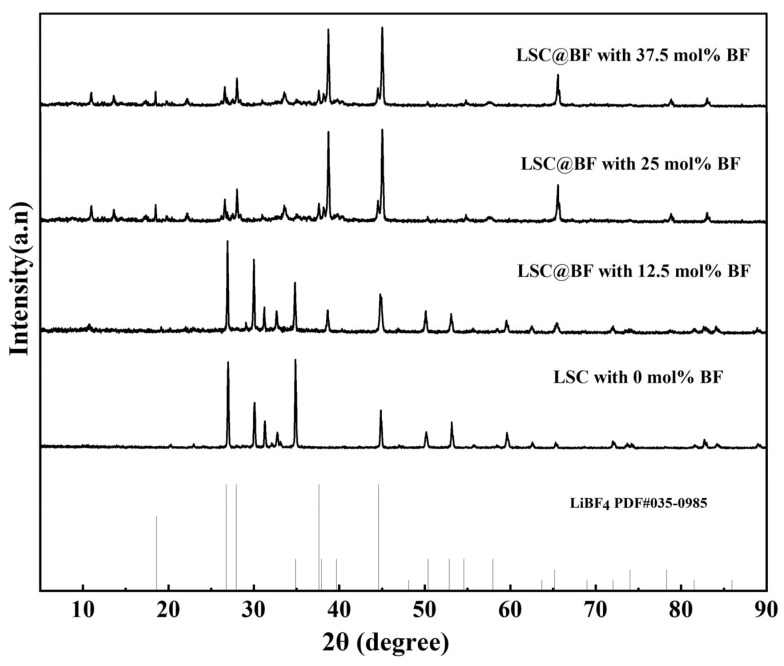
A comparison of the XRD patterns of pristine LSC and LSC@BF.

LSC@BF was prepared via solid-state sintering at 290–296 °C. XRD was used to assess the effect of LiBF_4_ incorporation on the crystal structure of the LSC. Figure 2 compares the diffraction patterns of pristine LSC, LiBF_4_ (PDF#035-0985), and LSC@BF. The characteristic peaks of both LSC and LiBF_4_ are clearly retained in the composite, indicating the absence of any solid-state reaction or phase transformation during sintering [26]. Furthermore, no detectable peak shift is observed for either component, ruling out lattice substitution or the formation of a solid solution [27].

The retention of characteristic diffraction peaks from both LSC and LiBF_4_ confirms that the composite consists of a mixture of the two phases rather than a newly formed compound. Additionally, no distinct diffraction peaks corresponding to LiF (a potential decomposition product of LiBF_4_) are observed, indicating negligible thermal decomposition of LiBF_4_ under the synthesis conditions. Compared to pristine LSC, LSC@BF exhibits slightly reduced peak intensities and minor peak broadening, suggesting partial amorphization induced during the LSC@BF preparation [27].

### 3.2. Morphology and Elemental Distribution Analysis

**Figure 3:**
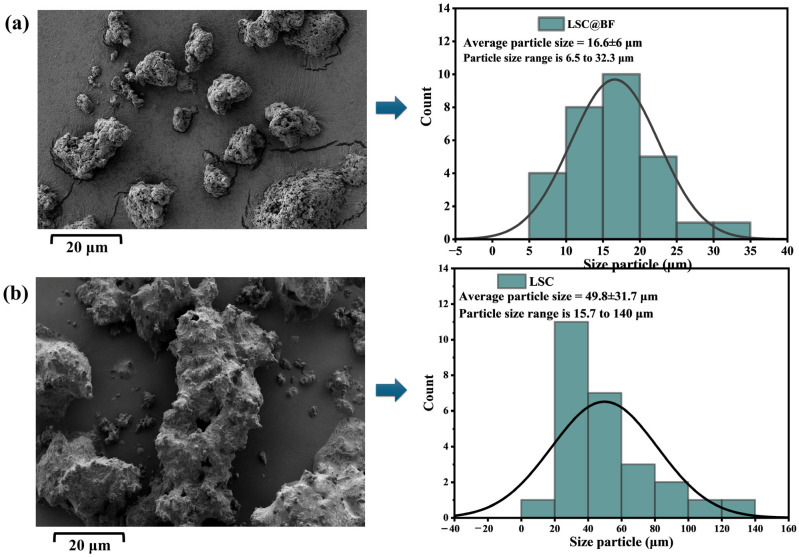
FESEM images for (**a**) LSC@BF and (**b**) LSC SSEs with their corresponding particle size distribution histograms.

The morphology and elemental distribution of LSC@BF and LSC powders were analyzed by FESEM and EDS mapping as shown in Figure 3. It can be observed that the LSC@BF consists of irregularly shaped particles with a slightly smooth and an average size of 16.6 ± 6 μm (Figure 3a), which is significantly smaller than that of the pristine LSC (49.8 ± 31.7 μm), exhibiting a slightly rough surface morphology (Figure 3b). The reduction in particle size is attributed to the mechanical milling process used to mix LSC with LiBF_4_. The smaller particles of LSC@BF can fill grain boundary gaps between neighboring particles, and the resulting stacked microstructure offers multiple pathways for lithium-ion transport [23]. This can further be illustrated by the intimate contact and non-porous morphologies between adjacent particles in cold-pressed LSC@BF pellet cross-section when compared with that of pristine LSC as shown in Figure S2a. The intimate contact between adjacent particles can further be useful in lithium dendrite penetration suppression [20,23].

The elemental distribution of LSC@BF mixture and LSC are studied by EDS mapping (Figures S3 and S4). It is clear that uniform distribution of S, Cl, and F elements can be observed both in LSC@BF mixture and LSC, which confirms the compositional homogeneity of LSC@BF mixture and LSC. The elemental homogeneity in LSC@BF and LSC SSEs ensures stable ionic transport, as it is a prerequisite for continuous Li-ion conduction pathways [20,26].

### 3.3. Ionic Transport

**Figure 4:**
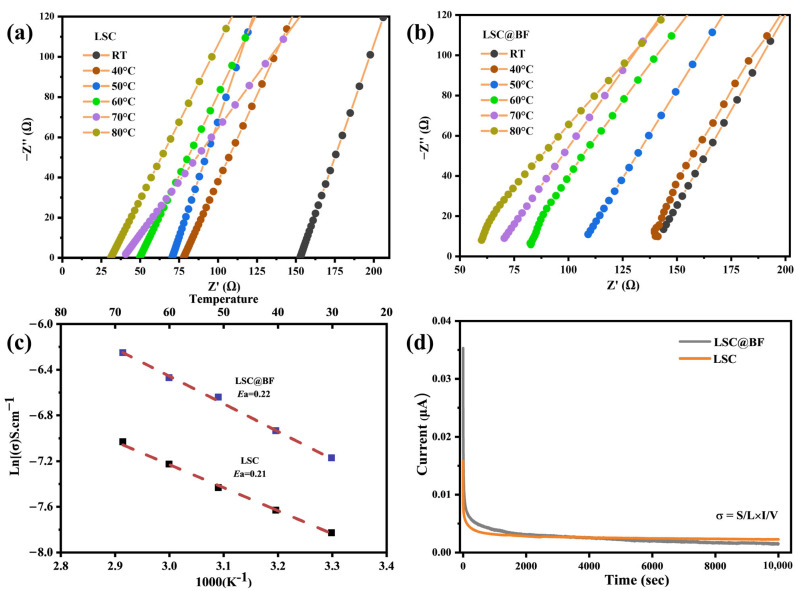
Nyquist plots of (**a**) LSC; (**b**) LSC@BF; (**c**) their Arrhenius plots and (**d**) direct current polarization plots for electronic conductivity measurement.

For LSC, the bulk ionic conductivity is 4.44 × 10^−4^ S cm^−1^ at room temperature, increasing to 8.69 × 10^−4^ S cm^−1^ at 40 °C, 9.84 × 10^−4^ S cm^−1^ at 50 °C, 1.36 × 10^−3^ S cm^−1^ at 60 °C, 1.66 × 10^−3^ S cm^−1^ at 70 °C, and 2.17 × 10^−3^ S cm^−1^ at 80 °C, as estimated from Figure 4a. After mixing and sintering with LiBF_4_, the resulting LSC@BF maintains high ionic conductivity values of 4.32 × 10^−4^, 4.44 × 10^−4^, 5.69 × 10^−4^, 7.52 × 10^−3^ 8.83 × 10^−3^, and 1.04 × 10^−3^ S cm^−1^ at room temperature, 40 °C, 50 °C, 60 °C, 70 °C, and 80 °C, respectively (estimated from Figure 4b). These values are comparable to those of highly conductive thio-halides and other fluoroborate containing SSEs including our previous report (Table S1). Moreover, the activation energy (*E*_a_) shows no significant change, estimated to be 0.21 eV for LSC and 0.22 eV for LSC@BF (Figure 4c).

As observed, the ionic conductivity of LSC is slightly higher than that of LSC@BF. This can be attributed to the presence of Cl^−^, which is less electronegative and forms weaker Li-Cl bonds, enabling facile Li-ion transfer [21]. Consequently, LSC exhibits slightly better Li-ion conduction compared to that of LiBF_4_-containing LSC@BF SSE. In the latter, the tetrahedral geometry of the highly electronegative ${BF}_{4}^{-}$ pseudo anion [28] forms stronger Li-F bonds, hindering Li-ion migration [22,29]. This can further be illustrated by the Nyquist and Arrhenius plots in Figure S5 and the corresponding ionic conductivity values summarized in Table S2, which show that the ionic conductivity of LSC@BF decreases with increasing LiBF_4_ content. Nevertheless, at optimal LiBF_4_ content (25 mol%), the electronegativity effects largely offset each other, resulting in only a slight increase in *E*_a_, as illustrated in Figure 4c. Furthermore, the high ionic conductivity in both SSE samples can be attributed to the homogeneous elemental distribution revealed by the EDS analysis (Figures S4 and S5), which establishes continuous conductive and long-range pathways for lithium-ion conduction [20,26]. Additionally, the presence of a sulfide enhances ionic mobility due to its soft, polarizable lattices, and wide diffusion pathways, which lower the *E*_a_ for Li-ion transport [30].

### 3.4. The Effect of LiBF_4_ on Cycling Stability

**Figure 5:**
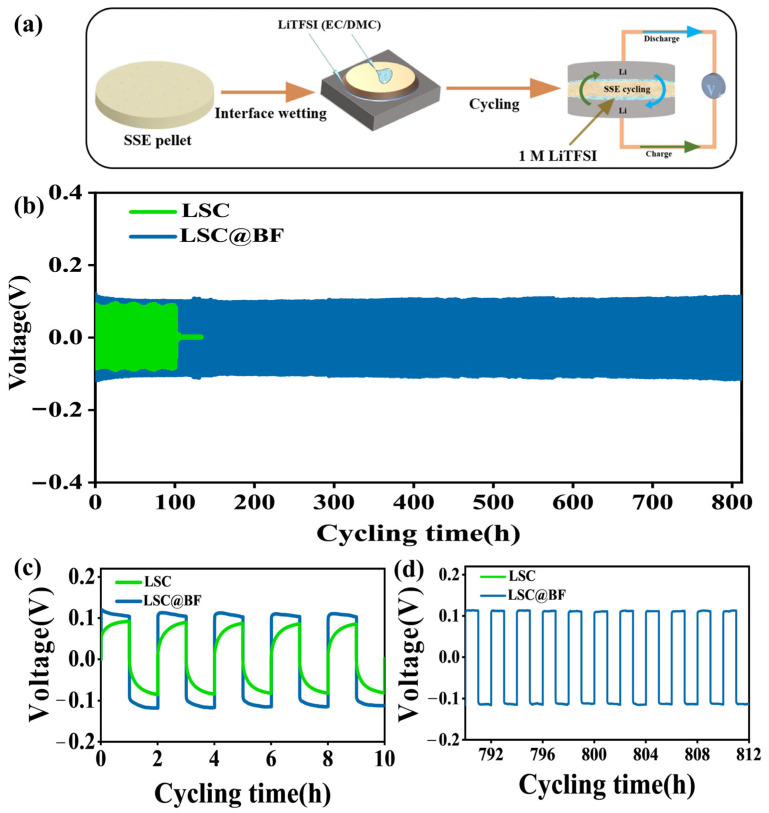
(**a**) Schematic preparation process of symmetric cells wetted with a trace of 1 M LiTFSI (EC/DMC) and plating/stripping working principle. (**b**) Li plating/stripping of LSC (green) and LSC@BF (blue) in Li symmetric cells at 0.2 mA cm^−2^. (**c**,**d**) Enlarged voltage profiles for LSC and LSC@BF cycling in Li symmetric cells during the initial stages, and for LSC@BF after 800 h of cycling time, respectively.

The electronic conductivities were also measured using DC polarization measurements, and the results are shown in Figure 4d. The LSC@BF SSEs sample exhibits the lowest electronic conductivity, approximately 8.51 × 10^−9^ S cm^−1^, compared to the pristine LSC SSE sample, which shows 1.42 × 10^−7^ S cm^−1^. In LMBs, SSEs with high electronic conductivity accelerate the electrochemical reaction between lithium and SSEs, thereby promoting lithium dendrite growth [31]. To confirm the correlation between high electronic conductivity in SSEs and lithium dendrite growth, symmetric cells were assembled and subjected to galvanostatic lithium plating/stripping cycling measurements at room temperature. However, the dry solid–solid interface inherently exhibits poor contact between metallic Li and the SSE, which severely limits battery operation. To improve contact of the Li/LSC@BF SSE interface, a trace of a 1 M LiTFSI (EC/DMC) wetting agent was introduced between the lithium metal electrode and the SSE, as shown in Figure 5a. Figure 5b shows the voltage profiles of lithium symmetric cells using pristine LSC or LSC@BF SSE samples during lithium plating and stripping under 0.2 mA cm^−2^ current densities. At the current density of 0.2 mA cm^−2^, the plating/stripping overpotentials of the cells containing pristine LSC as SSE is maintained below 0.13 V until it shorts after 100 h of cycling (Figure 5b, green curve), likely due to dendrite penetration through the LSC SSE [32,33], which is consistent with the measured electronic conductivity (Figure S4, yellow curve). In comparison to pristine LSC, the Li plating/stripping overpotentials of the LSC@BF mixture are slightly higher than that of LSC, but the cell maintains cycling stability for more than 800 h, surpassing that of LSC SSE by more than a factor of 8 (Figure 5b, blue curve).

The low overpotentials for LSC (inset in Figure 5c, green curve) can be ascribed to efficient Li-ion transport triggered by synergistic effect of Cl and S, which are known to facilitate ionic conductivity [34,35]. The stable cycling performance without degradation throughout the entire cycling period (Figure 5c,d insets), correlates positively with the measured electronic conductivity of LSC@BF, where a lower electronic conductivity suppresses lithium dendrite formation and promotes lithium deposition on the Li anode [31,36]. This behavior is primarily attributed to the synergistic effects between the highly conductive LSC and LiBF_4_, which effectively lower the interfacial charge-transfer resistance, as it is a known property of LiBF_4_ when used as liquid electrolyte additive in Li-ion batteries [37]. In such cases, LiBF_4_ slightly decomposes to release fluorine, which reacts with Li to form a LiF-rich, electronically insulated, and mechanically robust solid-electrolyte interphase (SEI) passivating layer, thereby suppressing lithium dendrite formation and growth.

In addition to possessing significantly improved interfacial stability at a current density of 0.2 mA cm^−2^, LSC@BF also demonstrates excellent cyclic performance at a slightly higher current density. As shown in Figure S6, LSC@BF can cycle stably for over 120 h at a current density of 1 mA cm^−2^, maintaining an areal capacity of 1 mAh cm^−2^ throughout the cycling duration. This result underscores its outstanding electrochemical stability and further confirms the enhanced interfacial compatibility with bare lithium metal electrodes. Notably, this excellent interfacial performance was achieved without the use of any protective coatings or lithium metal electrode alloying strategies, which are commonly employed in other studies on sulfide-containing SSEs.

### 3.5. In-Depth Mechanistic Analysis of the Solid-Electrolyte Interphase Formation

To elucidate the evolution of the Li|SSE interface and the formation of an interphase during cycling, EIS measurements were performed on symmetric cells assembled with LSC or LSC@BF (Figure S7). Before and after 50 h and 100 h of cycling, LSC exhibits a significant change in impedance, whereas LSC@BF shows only a minimal increase in total interfacial resistance throughout the testing time, as shown in Figure S7a and Figure S7b, respectively.

The increasing interfacial resistance in the LSC-based indicates continuous interfacial reactions between lithium metal and the SSE [38], leading to severe degradation and eventual short-circuiting, which is consistent with the observations in Figure 5. In contrast, the negligible interfacial resistance increase in the LSC@BF-based cell is attributed to the formation of a LiF-rich SEI. This LiF-rich layer is responsible for the markedly improved cycling performance observed in Figure 5. Previous studies have extensively discussed fluorinated lithium salts, such as LiBF_4_, as fluorine sources for the sacrificial decomposition-induced formation of a LiF-rich SEI, which is closely associated with enhanced cycling performance in LMBs [39,40,41].

**Figure 6:**
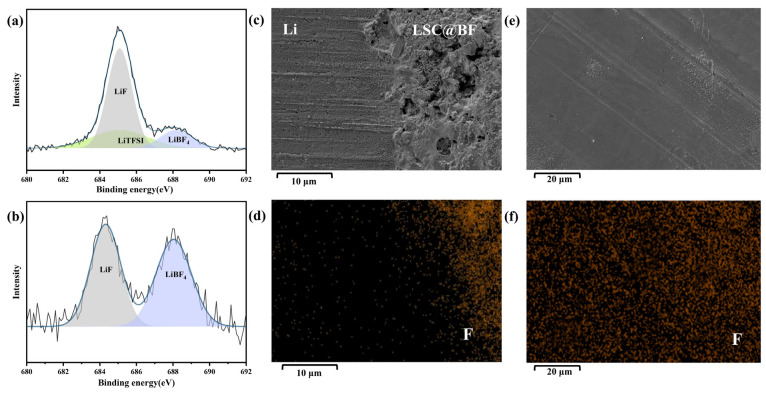
XPS spectra of the Li metal surface after cycling with LSC@BF SSE (**a**) with and (**b**) without LiTFSI (EC/DMC) liquid electrolyte. (**c**) Cross-sectional FESEM image of the Li|LSC@BF interface after 100 h of cycling and (**d**) its corresponding fluorine EDS map. (**e**) Surface FESEM image of the cycled Li metal electrode and (**f**) its corresponding fluorine EDS map.

To confirm the formation of a LiF-rich passivating interphase layer from LiBF_4_, XPS was performed on the surface of the post-cycled Li metal electrode. The F 1s spectrum of the sample from the cell containing the LiTFSI wetting agent (Figure 6a) shows two distinct peaks: a peak at 685 eV with a shoulder, attributed to LiF [42,43], where the shoulder corresponds to reduction of LiTFSI originating from the liquid electrolyte wetting agent [43]. There was another peak at 688.5 eV, assigned to residual LiBF_4_ salt from the LSC@BF SSE [44]. To rule out the possibility that the LiF signal arise solely from the LiTFSI wetting agent, a control sample cycled without LiTFSI was also analyzed by XPS (Figure 6b). The presence of the characteristic LiF peak at 684.8 eV in this control sample provides definitive evidence that the peak at 685 in Figure 6a originates from the interaction of LSC@BF SSE and the Li metal electrode, confirming that LiBF_4_ in LSC@BF undergoes sacrificial decomposition to form a LiF-rich SEI. Notably, the formation of a LiF-rich SEI requires a fluorine source, and no fluorine is present in EC/DMC, as has been discussed by Chen’s group [45].

**Figure 7:**
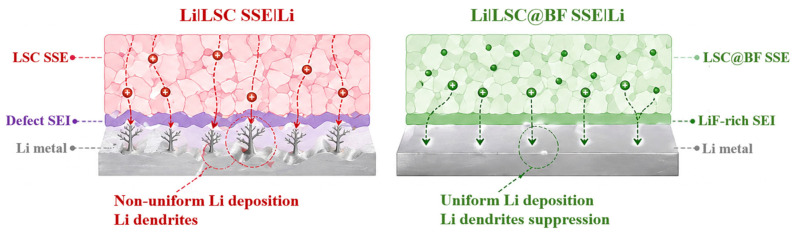
Schematic comparison for SEI formation between pristine LSC (which causes non-uniform Li deposition, defective SEI, and dendrite growth) and LSC@BF (which promotes uniform Li deposition and a robust LiF-rich SEI, suppressing dendrites for a stable interface).

To further understand the interfacial stabilization mechanism enabled by mixing and sintering LSC with LiBF_4_, FESEM combined with EDS techniques were employed to examine the interfaces of symmetric cells containing either pristine LSC or LSC@BF mixture. After cycling with bare Li metal for 100 h at a current density of 0.1 mA cm^−2^, cross-sectional morphological and elemental distribution analyses were performed (Figure 7 provides a schematic comparison of the two cases). In contrast to the FESEM images of the Li|LSC interface, which show evidence of lithium dendrite formation (Figure S8), the Li|LSC@BF interface images reveal a dense, void-free contact layer formed between the lithium metal and the electrolyte (Figure 6c). The absence of dendrite penetration and the minimal gaps at the interface suggest a stable interphase layer and favorable interfacial contact, which likely accounts for the observed improvement in cyclic stability [46]. Corresponding EDS elemental mapping (Figure 6d) shows an enrichment of fluorine localized at the Li|SSE interface for LSC@BF, consistent with the formation of a fluorine-containing interphase during cycling [23].

In addition, the surfaces of the cycled Li metal electrodes were also examined by FESEM-EDS after cycling for 100 h with LSC or LSC@BF and compared with the surface of the fresh Li metal (Figure S9). The surface of the fresh lithium metal is relatively smooth (Figure S9a). After 100 h of continuous cycling with LSC, the lithium metal surface exhibits severe dendritic features characterized by extensive roughening and irregular deposits (Figure S9b). Continuous interfacial side reactions prevent the homogeneous deposition of lithium ions during cycling [47,48], leading to excessive local stresses at the interface and eventually destruction of the interfacial SEI. The dendritic features can be further attributed to the porous microstructure of the pelletized LSC (Figure S2b), which readily allows dendrites to grow through its pores and cracks, ultimately causing short-circuiting [38]. In contrast, after 100 h of continuous cycling with LSC@BF, the lithium metal surface remains dense and uniform, with no visible dendritic features or pulverization (Figure 6e,f). The continuous, crack-free structure observed after 100 h of plating/stripping confirms the dendrite-suppressing behavior [23] of the LSC@BF SSE, which arises from: (i) the formation of a mechanically robust LiF-rich SEI [38], evidenced by the uniform F distribution on the post-cycling Li metal surface (Figure 6f), and (ii) a robust physical barrier provided by the dense microstructure of the pelletized LSC@BF SSE (Figure S2a), which resists dendrite penetration [20,49]. These FESEM results are consistent with the excellent cycling performance of Li metal symmetric cell shown in Figure 5.

## 4. Conclusions

This work reports a fluorine-modified thio-halide SSE, LSC@BF, prepared via a two-step straightforward solid-state method by incorporating LiBF_4_ into Li_3_SCl. XRD analysis confirms that the obtained SSE consists of a mixture of Li_3_SCl and LiBF_4_ without the formation of new crystalline phases, while slight peak broadening and intensity reduction suggest partial amorphization. Electrochemical characterization demonstrates that the optimizing the content of LiBF_4_ in LSC@BF effectively balances the trade-off between ionic conductivity and interfacial stability: although the ionic conductivity shows only a slight decrease, the interfacial compatibility with lithium metal is significantly enhanced. The optimized LSC@BF SSE delivers stable lithium plating/stripping for over 800 h at 0.2 mA cm^−2^ and maintains improved performance at higher current densities, far exceeding that of pristine LSC. This enhanced stability is attributed to the formation of a LiF-rich interphase at the SSE|Li metal interface, as supported by XPS and post-cycling FESEM-EDS morphological analysis. While LSC@BF SSE demonstrates compelling electrochemical performance, including high ionic conductivity, low activation energy, and stable compatibility with bare lithium metal, further studies are necessary to fully realize its practical potential. In particular, extending to full cell configurations is essential, including cathode compatibility studies and composite cathode formulation, represents a critical next step toward practical application. Moreover, the use of advanced in situ characterization techniques will be critical to directly capture the formation and evolution of the LiF-rich SEI, providing mechanistic insights that cannot be resolved through ex situ analysis alone.

## Data Availability

The original contributions presented in this study are included in the article/Supplementary Material. Further inquiries can be directed at the corresponding author.

## Supplementary Materials

The following supporting information can be downloaded at https://www.mdpi.com/article/10.3390/molecules31132313/s1, Figure S1. (a) Diameter and (b) thickness of the typical cold-pressed LSC@BF solid-electrolyte pellet. Figure S2. FESEM images of pelletized (a) LSC@BF and (b) LSC. Figure S3. EDS elemental mapping images of LSC@BF SSE. Figure S4. EDS elemental mapping images of LSC SSE. Figure S5. EIS Nyquist plots of LSC@BF SSE at higher LiBF_4_ content, and their corresponding Arrhenius plots. Figure S6. Li plating/stripping of LSC@BF in Li symmetric cells cycled at 1 mA cm^−2^. Figure S7. Nyquist plot before and after cycling for 100 h in a cell using (a) LSC and (b) LSC@BF. Figure S8. The cross-section FESEM image of the Li|LSC interface after cycling for 100 h at 0.1 mA cm^−2^. Figure S9. FESEM images of (a) fresh Li metal surface morphology, (b) Li metal surface morphology after cycling for 100 h with LSC at 0.1 mA cm^−2^. Table S1. Ionic transport comparison of our SSEs with other thio-halides and other fluorinated SSEs at room temperature. Table S2. Ionic conductivity and activation energy values comparison of LSC@BF SSEs with different LiBF_4_ content tested at room temperature (RT), 50 °C and 70 °C. References [50,51,52,53,54] are cited in the Supplementary Materials.
