# Supplementary material for: Interfacial Tuning of Sulfohalide Electrolytes by LiBF4 for Stable Lithium Metal Batteries
Source: Molecules. 2026 Jul 1;31(13):2313. doi: 10.3390/molecules31132313 (PMC13363403; doi:10.3390/molecules31132313)
Supplement: Supplementary file 1 [file molecules-31-02313-s001.zip › molecules-4342942-supplementary.pdf]

Interfacial Tuning of Sulfohalide Electrolytes by $LiBF_4$ for Stable Lithium Metal Batteries

Peng Tang*[1], John Prochest Kachenje[1], Zhengle Xiang[2], Dachun Wang[2], Yanyi Tao[1], Peng Yang[1], Huihui Li[1], Xiaoping Qin[1], Song Qing[1], Wei Cao[1], Qinyu Chen[1], Yongmin Wu*[3], Haiyang Tian[1]

[1]School of Chemical Engineering, Sichuan University of Science and Engineering, Zigong 643000, Sichuan, China, *E-mail: tpdzyyx@163.com. [2]Clean Energy Branch of China National Offshore Oil Corporation Energy Technology & Services Co., Ltd., 293 Tangubohaishiyou Road, Tianjin 300450, China. [3]State Key Laboratory of Space Power-sources Technology, Shanghai Institute of Space Power Sources, 2965 Dongchuan Road, Shanghai 200245, China.

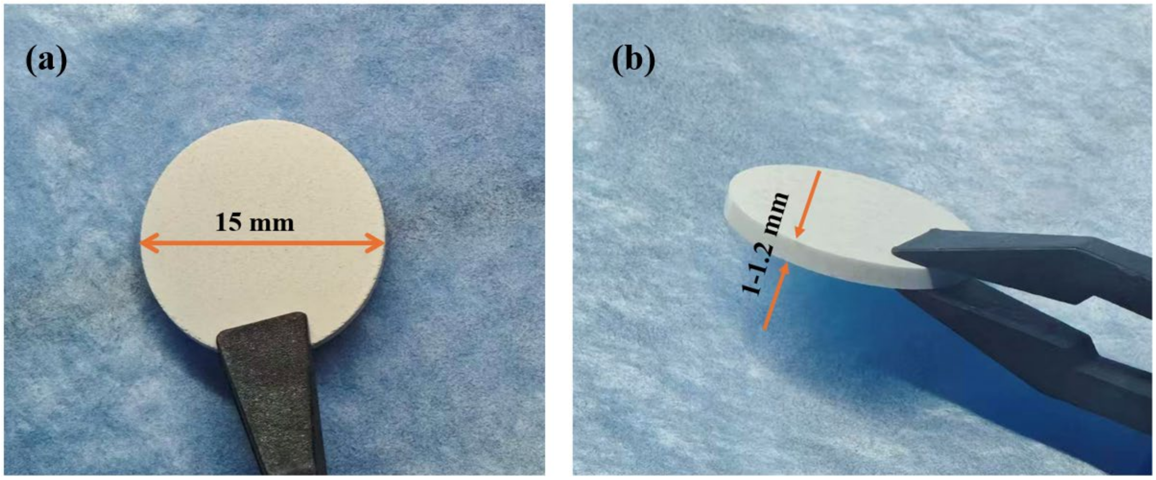

Figure S1. (a) Diameter and (b) thickness of the typical cold-pressed LSC@BF solid electrolyte pellet.

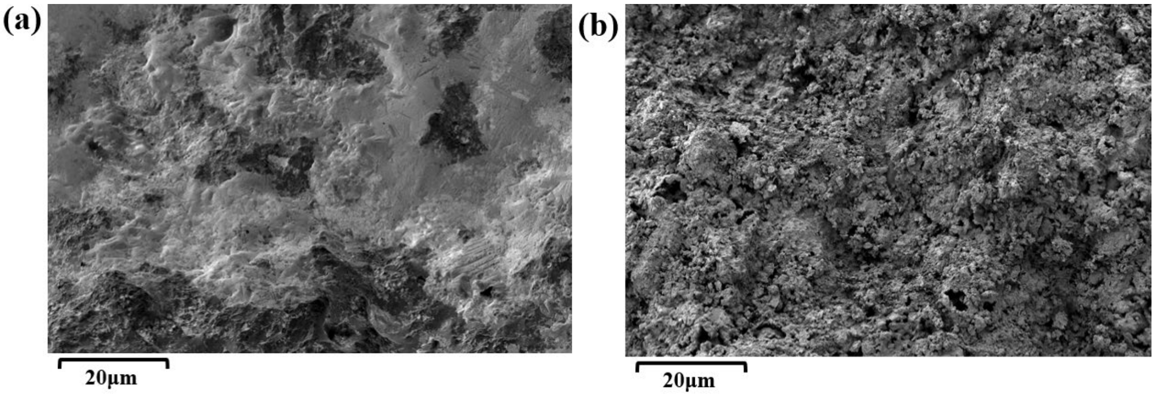

Figure S2. FESEM images of pelletized (a) LSC@BF and (b) LSC.

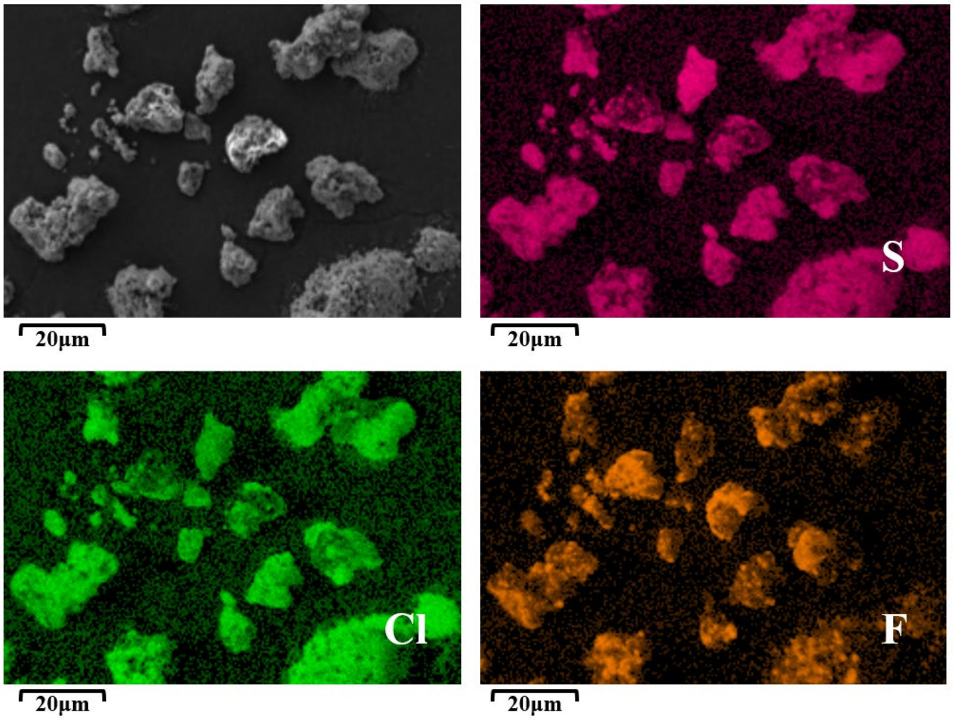

Figure S3. EDS elemental mapping images of LSC@BF SSE.

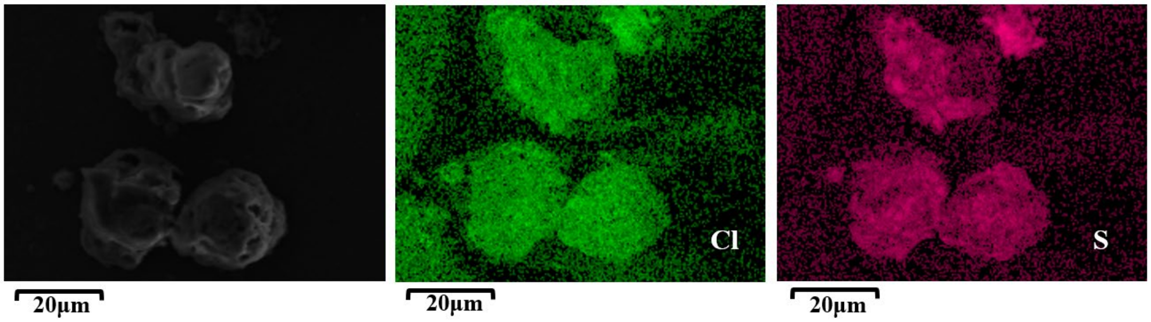

Figure S4. EDS elemental mapping images of LSC SSE.

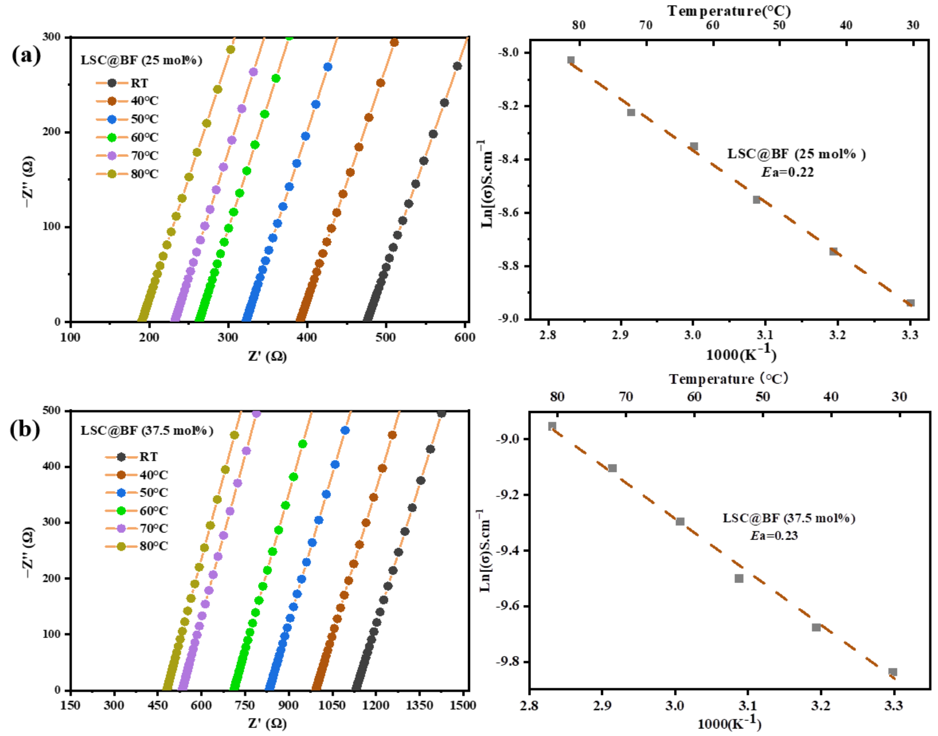

Figure S5. (a) EIS Nyquist plots of LSC@BF SSE at higher $LiBF_4$ content, and their corresponding Arrhenius plots.

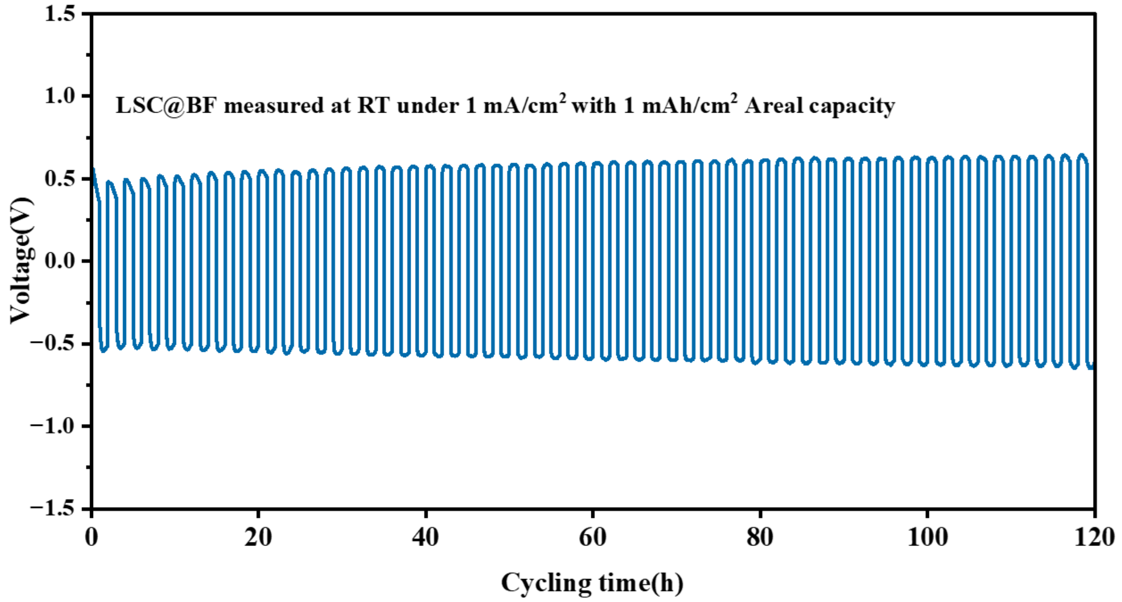

Figure S6. Li plating/stripping of LSC@BF in Li symmetric cells cycled at 1 mA cm^{-2}.

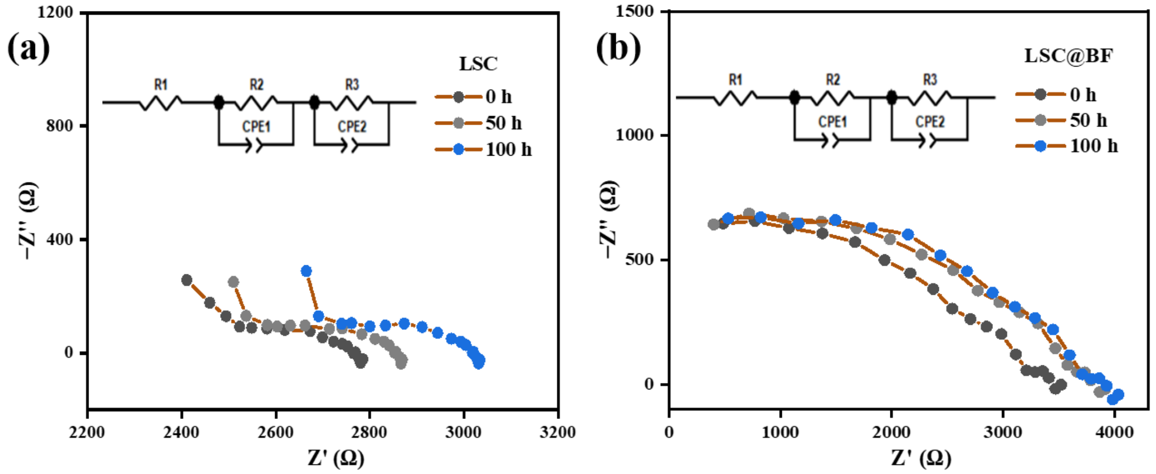

Figure S7. Nyquist plot before and after cycling for 100 h in a cell using (a)LSC and (b)LSC@BF.

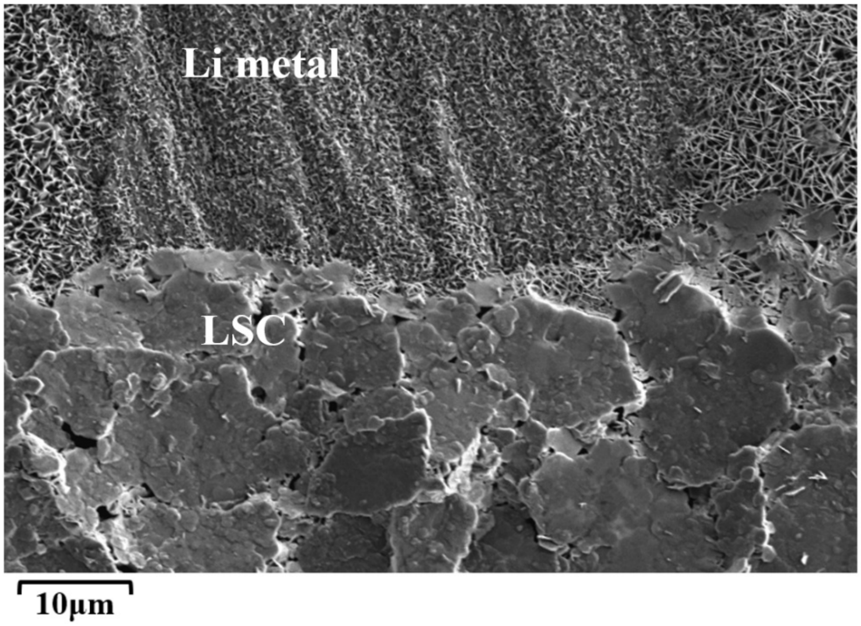

Figure S8. The cross-section FESEM image of the Li|LSC interface after cycling for 100 h at 0.1 mA cm^{-2}.

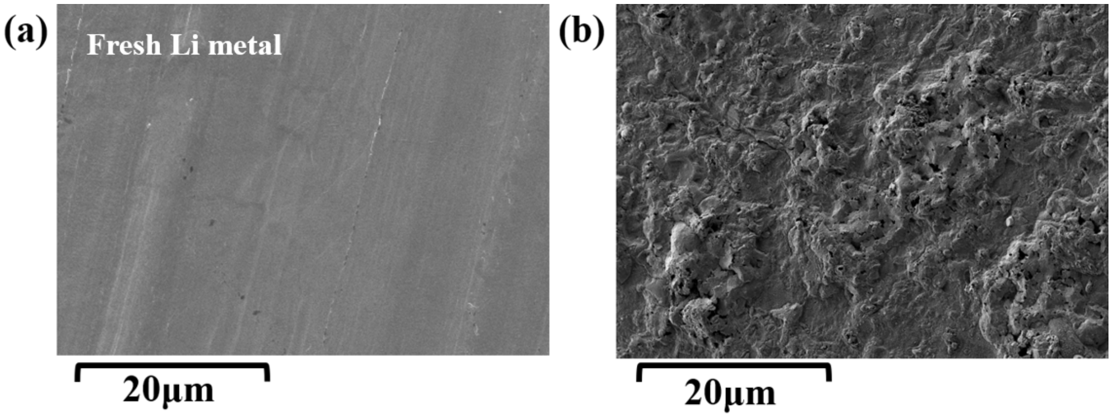

Figure S9. FESEM images of (a) fresh Li metal surface morphology, (b) Li metal surface morphology after cycling for 100 h with LSC at 0.1 mA cm^{-2}.

Table S1. Ionic transport comparison of our SSEs with other thio-halides and other fluorinated SSEs at room temperature.

**SSEs**	**Bulk ionic conductivity ($S\ cm^{-1}$)**	**Activation energy (eV)**	**References**
LSC	4.44×10^{-4}	0.21	This work
Li_6PS_5Cl	4.96×10^{-3}	0.34	Ref. [1]
Li_4SnS_4	7.0×10^{-5}	0.41	Ref. [2]
$Li_7Ge_3PS_{12}$	1.1×10^{-4}	0.26	Ref. [3]
LSC@BF	4.32×10^{-4}	0.22	This Work
$LSCF_{0.25}$	3.38×10^{-4}	0.23	Ref [4]
$LiBH_4$@$LiBF_4$	0.9×10^{-5}	No report	Ref. [5]
LAGP@$LiBF_4$	3.21×10^{-4}	0.37	Ref. [6]
LATP@$LiBF_4$	8.5×10^{-4}	No report	Ref. [7]
$Li_4(BH_4)_3I$@SBA-15	2.5×10^{-4}	0.46	Ref. [8]
$LiBF_4$@Li_2S@LiCl	1.19×10^{-6}	0.28	Ref. [9]

Table S2. Ionic conductivity and activation energy values comparison of LSC@BF SSEs with different $LiBF_4$ content tested at room temperature (RT), 50℃ and 70℃.

$LiBF_4$ in LSC@BF (mol%)	Ionic conductivity (S cm^{-1})		Activation Energy (eV)
0	RT	1.28×10^{-5}	0.21
	50℃	1.95×10^{-5}	
	70℃	2.22×10^{-5}	
12.5	RT	3.87×10^{-5}	0.22
	50℃	5.70×10^{-5}	
	70℃	8.89×10^{-5}	
25	RT	7.61×10^{-5}	0.22
	50℃	9.67×10^{-5}	
	70℃	1.15×10^{-4}	
37.5	RT	2.62×10^{-4}	0.23
	50℃	5.88×10^{-4}	
	70℃	9.30×10^{-4}	

References

[1] Ganapathy, C.; Yu, S.; Hageman, J.; Van Eijck, L.; Van Eck, E.R.; Zhang, L.; Schwietert, T.; Basak, S.; Kelder, E.M.; Wagemaker, M. Facile synthesis toward the optimal structure-conductivity characteristics of the argyrodite Li_6PS_5Cl solid-state electrolyte, *ACS applied materials & interfaces*, **2018**,10, 33296-33306.

[2] Kaib, T.; Haddadpour, S.; Kapitein, M.; Bron, P.; C.; Schröder, Eckert, H.; Roling, B.; Dehnen, S. New lithium chalcogenidotetrelates, LiChT: synthesis and characterization of the Li^+-conducting tetralithium ortho-sulfidostannate Li_4SnS_4, *Chemistry of Materials*, **2012**, 24, 2211-2219.

[3] Inoue, Y.; Suzuki, K.; Matsui, N.; Hirayama, M.; Kanno, R. Synthesis and structure of novel lithium-ion conductor $Li_7Ge_3PS_{12}$, *Journal of Solid State Chemistry*, **2017**, 246, 334-340.

[4] Kachenje, J. P. Tang, P.; Tao, Y.Y.; Yang, P.; Qin, X.P.; Qing, S.; Cao, W.; Gong, S.H.; Chen, Q.Y.; Zhang, L.; Lv, X.M.; Liu, Q.X.; Liu, J.L.; Zhao, X.Y. Tuning bulk ionic transport and interfacial reactivity in sulfohalide solid electrolytes via controlled fluorination, *Journal of Energy Storage*, **2026**, accepted.

[5] Kort, L.M.; Gulino, V.; Blanchard, D.; Ngene, P. Effects of $LiBF_4$ Addition on the Lithium-Ion Conductivity of $LiBH_4$, *Molecules*, **2022**, 27, 2187.

[6] Gao, C.; Zhou, J.; Zhang, Q.; Cui, P.; Zhang, Q.; Wei, W. Influence of $LiBF_4$ sintering aid on the grain boundary and conductivity of LAGP electrolyte, *Functional Materials*

Letters, **2023**, 16, 2350001.

[7] Dai, L.; Wang, J.; Shi, Z.; Yu, L.; Shi, J. Influence of $LiBF_4$ sintering aid on the microstructure and conductivity of LATP solid electrolyte, *Ceramics International*, **2021**, 47, 11662-11667.

[8] Yin, L.; Yuan, H.; Kong, L.; Lu, Z.; Y. Zhao, Engineering Frenkel defects of anti-perovskite solid-state electrolytes and their applications in all-solid-state lithium-ion batteries, *Chemical Communications*, **2020**, 56, 1251-1254.

[9] Tang, P.; Kachenje, J.P.; Yang, P.; Qin, X.; Cao, W.; Tao, Y.; Heng, D.; Gong, S.; Chen, R.; Chen, Q. Enhanced ionic mobility and cycling performance of $LiBF_4$-based inorganic solid-state electrolyte for solid state Lithium metal batteries, International Conference on Optics, Electronics, and Communication Engineering (OECE 2025), *SPIE*, **2025**, 1278-1288.
